# Developing a patient-centered outcome measure for complementary and alternative medicine therapies II: *Refining content validity through cognitive interviews*

**DOI:** 10.1186/1472-6882-11-136

**Published:** 2011-12-29

**Authors:** Jennifer J Thompson, Kimberly L Kelly, Cheryl Ritenbaugh, Allison L Hopkins, Colette M Sims, Stephen J Coons

**Affiliations:** 1 Division of Biological Sciences, University of Georgia, Athens, GA, USA; 2 Department of Family and Community Medicine, School of Medicine, University of Arizona, Tucson, AZ, USA; 3 School of Anthropology, University of Arizona, Tucson, AZ, USA; 4 Patient-Reported Outcome Consortium, Critical Path Institute, Tucson, AZ, USA

**Keywords:** Complementary and alternative medicine (CAM), patient-reported outcomes (PROs), cognitive interviewing, patient-centered care, non-specific outcomes, questionnaire development, retrospective pre-test, well-being

## Abstract

**Background:**

Available measures of patient-reported outcomes for complementary and alternative medicine (CAM) inadequately capture the range of patient-reported treatment effects. The Self-Assessment of Change questionnaire was developed to measure multi-dimensional shifts in well-being for CAM users. With content derived from patient narratives, items were subsequently focused through interviews on a new cohort of participants. Here we present the development of the final version in which the content and format is refined through cognitive interviews.

**Methods:**

We conducted cognitive interviews across five iterations of questionnaire refinement with a culturally diverse sample of 28 CAM users. In each iteration, participant critiques were used to revise the questionnaire, which was then re-tested in subsequent rounds of cognitive interviews. Following all five iterations, transcripts of cognitive interviews were systematically coded and analyzed to examine participants' understanding of the format and content of the final questionnaire. Based on this data, we established summary descriptions and selected exemplar quotations for each word pair on the final questionnaire.

**Results:**

The final version of the Self-Assessment of Change questionnaire (SAC) includes 16 word pairs, nine of which remained unchanged from the original draft. Participants consistently said that these stable word pairs represented opposite ends of the same domain of experience and the meanings of these terms were stable across the participant pool. Five pairs underwent revision and two word pairs were added. Four word pairs were eliminated for redundancy or because participants did not agree on the meaning of the terms. Cognitive interviews indicate that participants understood the format of the questionnaire and considered each word pair to represent opposite poles of a shared domain of experience.

**Conclusions:**

We have placed lay language and direct experience at the center of questionnaire revision and refinement. In so doing, we provide an innovative model for the development of truly patient-centered outcome measures. Although this instrument was designed and tested in a CAM-specific population, it may be useful in assessing multi-dimensional shifts in well-being across a broader patient population.

## Background

Patients receiving complementary and alternative medicine (CAM) therapies often report experiencing effects beyond those associated with their specific treatment goals, including unanticipated outcomes and multi-dimensional shifts in overall well-being, energy, clarity of thought, emotional and social functioning, lifestyle patterns, inner life, and spirituality [1-11]. This research project aimed to develop an instrument to measure these 'emergent outcomes' of treatment--that is, those outcomes that may be beyond the direct biomedical endpoints for which patients sought therapy, and may or may not have been part of the 'expected' outcomes from the perspective of CAM providers [See [12]].

Patient-reported outcomes (PROs), like those captured in this instrument, document subjective states of health and illness, including symptoms, function, and quality of life, and measures of PROs are increasingly being employed in an effort to demonstrate the efficacy of health interventions and market health products [13,14]. In developing and refining this instrument, we used patients' experiences and language to capture and measure outcomes that may be important to patients but often go unnoticed or even dismissed by clinicians and researchers. Although our instrument is, by definition, assessing PROs, we refer to it as a patient-centered outcome measure based on the extensive patient involvement in its development.

Phase 1 of the project, reported in greater detail in Ritenbaugh et al. [12], identified relevant concepts to be measured. The initial phase of the project (Phase 1a), involved the secondary analysis of interviews with individuals who reported transformational experiences with CAM and other mind-body therapies. At that stage, the research team focused on identifying exemplar phrases from participants' descriptions of their experiences. Phase 1b consisted of 'evocative interviews' in which a new sample (composed of individuals who had experienced significant shifts in well-being following CAM use) took part in an extensive 'think aloud' activity to determine which phrases (from 1a) were the most relevant and accurate descriptions of their personal experiences. Phase 1c involved developing the initial format and content of the questionnaire, in which the research team identified the most highly endorsed phrases from evocative interviews and, from these phrases, created 18 word pairs for the initial version of the Self-Assessment of Change questionnaire.

The concepts identified in Phase 1 were refined in Phase 2, reported here, in which we evaluated five versions of the questionnaire through in-depth cognitive interviews with individuals who had experienced a broad range of shifts in well-being following CAM and other mind-body therapies. In these interviews, we paid close attention to the meanings participants ascribed to the terms on the questionnaire, whether these meanings were consistent across participants, whether participants felt that terms paired together were representative of positive and negative endpoints of the same domain of lived-experience, and whether participants were able to indicate their experience on the scales at two points in time. These data guided our revisions to the questionnaire.

Whereas Ritenbaugh et al. [12] documents the patient-centered generation of items on the initial draft of this questionnaire (Phases 1a-1c), this paper focuses on assuring the content validity of the questionnaire. As described by the US Food and Drug Administration [13], content validity is supported by evidence from qualitative research demonstrating that the instrument measures the concepts of interest — including documentation that the items and domains of an instrument are appropriate and comprehensive relative to its intended measurement concept, population, and use. Hence, this paper emphasizes patient-involvement in the iterative process of questionnaire refinement to ensure that the items included on the final questionnaire were appropriate, comprehensive, and well-understood by our target population of individuals using CAM and other mind-body therapies (Phase 2). To demonstrate content validity and to provide detailed information for researchers using the questionnaire in their own projects, we detail the derivation of items on the Self-Assessment of Change questionnaire, describe the concepts being measured, provide exemplar quotes of each concept in participants' own words, and demonstrate that respondents understood the questionnaire, in terms of both content and process.

## Methods

### Self-Assessment of Change Questionnaire

As described elsewhere, the Self-Assessment of Change questionnaire (available at http://www.selfassessmentofchange.org) was designed to systematically assess a broad range of shifts in well-being across CAM systems, therapeutic modalities, and conditions [12]. While completing the self-administered questionnaire, respondents are asked to "reflect on life changes" that they have experienced since *[beginning a CAM therapy]*. The instructions on the questionnaire leave the area in brackets blank so that clinicians or researchers can tailor the questionnaire to measure respondents' self-reported change in relation to the appropriate benchmark. Respondents are presented with a series of word pairs, the negative and positive poles of a shared domain of experience (e.g., *exhausted/energized, anxious/calm*), separated by a 100 mm visual analog scale. In order to measure change in these domains of experience over time, we opted for a retrospective pre-test format [15-18]. Respondents are instructed to mark each line to indicate where they were 'before' (B) *[the CAM therapy or other treatment] *and where they are 'now' (N). Examples included in the questionnaire instructions illustrate that participants can indicate varying degrees of positive change, no change, or negative change over time.

### Cognitive Interviews

Cognitive interviewing is a method used in questionnaire development to assess whether respondents comprehend and respond to questionnaire items in the way researchers intend them, and to provide information for questionnaire modification and improvement. Participants are asked to actively reflect upon and verbally articulate the process of responding to a questionnaire. This method is particularly useful in identifying unanticipated problems in the design of a questionnaire prior to its widespread use [19-21]. By coming at the problems from the respondents' perspective, cognitive interviews can help to pinpoint the trouble and elicit suggestions for how to fix it. Questionnaire revision using cognitive interviewing is an iterative process in which one or more revised versions of the questionnaire are subjected to cognitive interviews with small numbers of participants purposively selected because of their ability to offer relevant experience or insight [19,22]. Researchers have also used this method in developing and establishing content validity of instruments measuring PROs [13,22,23].

The cognitive interviews for this study were designed following the protocols described in Beatty and Willis [24] and Willis [19,25]. We employed 'verbal probe' and 'think aloud' techniques immediately after participants completed the entire questionnaire [19,24,26]. Drawing upon short term memory, this technique likely yields the same information as interviews in which participants verbalize their thought processes in 'real-time' as they complete the questionnaire. Our approach allowed us to minimize the distractions and influences the process may have on questionnaire responses themselves, [19,22,24] while providing us the opportunity to observe the process by which participants responded to the entire instrument.

Five interviewers conducted our cognitive interviews after receiving training by a member of the research team with experience designing and conducting cognitive interviews (SJC). Training consisted of a presentation that included issues particular to conducting cognitive interviews as compared to other interviewing styles, such as open-ended and semi-structured interviewing techniques. Interviewers then practiced conducting cognitive interviews with a partner or other members of the research team. Importantly, interviewers themselves role-played as study participants and were interviewed by another staff member or interviewer. This process was critical in helping interviewers understand how the cognitive interview is different from other types of interviews, what it is like to be a participant in a cognitive interview, and why it is essential to stay exactly with the script. It also helped interviewers develop techniques for explaining the interview style and eliciting responses from participants.

### Sample/Recruitment

Participants for this phase of research were selected from the Tucson, AZ and Vancouver, Canada areas. Men and women between the ages of 18-65 were eligible to participate in the interviews if they had experienced "significant changes, shifts, or transformations in their lives after using CAM therapies" (as described in the recruitment materials). These shifts were subjectively defined as something meaningful to the participant that included changes in physical, cognitive, emotional and/or spiritual domains of experience. They include the specific outcomes of the CAM treatment (such as reduced pain or increased relaxation), as well as broader shifts in well-being beyond the specific outcomes for which they sought CAM treatment, those we call 'emergent outcomes.' Participants were recruited via fliers posted at local health food stores, coffee shops, CAM practitioner offices, and local area email listserves. Interested individuals were asked to contact a member of the research staff who determined through a short conversation whether he/she fit the study inclusion and exclusion criteria--most importantly, whether the potential participant felt he/she had experienced a meaningful shift while using a CAM therapy.

Cognitive interviews were conducted with a multi-cultural sample of 4 men and 24 women. Participants self-identified as follows: White (n = 17); African-American/Black (n = 4); Hispanic (n = 3); Asian (n = 1); Canadian (n = 1); mixed-White and Asian (n = 1). One participant did not self-identify a category. While this represents a different sample from the original data set subjected to secondary analysis (Phase 1a), ten participants in the research phase described in this article (Phase 2) also participated in evocative interviews (Phase 1b) for this study. Participants had used a wide range of CAM and mind-body therapies including yoga, acupuncture, massage therapy, Reiki, naturopathy, and homeopathy in relation to broad range of illnesses and conditions, such as temporomandibular joint disorder, insomnia, polycystic ovarian disorder, cancer, HIV, migraines, anxiety, and depression.

### Data Collection

After receiving informed consent, interviewers gave participants the version of the questionnaire being tested and asked participants to read the instructions and rearticulate them to the interviewers. This was intended both to assess the clarity of instructions and to ensure that participants understood what they were being asked to do with the questionnaire. Any confusion as to how to complete the questionnaire was noted and clarified at this time. Participants then completed the questionnaire in writing while interviewers retreated from the interaction and unobtrusively observed how the participants went about completing the questionnaire. Following this activity, interviewers began the cognitive interview process with participants. Interviewers guided participants through each word pair on the questionnaire, systematically asking them to articulate:

*1. Whether the word pairs (e.g., exhausted/energized) were relevant to their individual experience*.

*2. Whether the terms on either end of the scale represented opposite ends of the same domain of experience*.

3. *How they interpreted each of the terms individually*.

*4. How they determined where to place the 'before' and 'now' marks on the line*.

Cognitive interviews averaged approximately 90 minutes. They were audio-recorded and transcribed verbatim. Two participants declined to complete the questionnaire, but completed a 'think aloud' version of the cognitive interview in which they discussed each item on the questionnaire in the same format described above.

### Data Analysis

Data collected from the cognitive interviews were analyzed in two stages: Stage One was part of the iterative process of questionnaire development in which participants' responses were assessed after each cognitive interview for indications of consensus or problems with regard to the cognitive interview questions above. The interviewer tracked participant responses during the interview on a standardized form. When two or more participants had similar problems or critiques, the research team made changes to the draft questionnaire, which was then tested in another round of cognitive interviews with three or more participants. During the later stages of the cognitive interview process, when it was clear to the investigators that consensus was being reached on a majority of word pairs, the decision was made to hold a team meeting immediately following each set of three cognitive interviews. During these meetings, interviewers presented data to the investigators about those terms or word pairs that study participants suggested they "didn't like," couldn't understand, or to which they suggested changes. The team then discussed these suggested changes, which included a review of phrases well-endorsed in the previous phase of the research project and previous versions of the questionnaire, to determine if there was a term or word pair that could be added or substituted on the questionnaire. These revisions were then tested again in at least three cognitive interviews. The cognitive interview process was concluded upon unanimous confirmation in interviews with three separate individuals that there were no changes they would suggest to the instrument. This 'real-time' process of analysis, revision, and re-testing was repeated until consensus among participants indicated that the terms, word pairs, and format of the questionnaire were well-understood. The details of this process are discussed in the results/discussion section below.

Stage Two of the analysis assessed the language and meaning that participants ascribed to the terms and word pairs on the final questionnaire. Following the completion of cognitive interviews and the finalization of the questionnaire, transcripts of cognitive interviews were imported into Atlas.ti http://www.atlasti.com, widely used software for qualitative coding and analysis. We developed our code list to analyze participants' responses to each of the four cognitive interview questions (above) for each word pair on the questionnaire. Codes were established for each of the sixteen word pairs on the final version of the questionnaire, terms that were eliminated, positive and negative valence (to represent either side of the scale), time frame ('before' and 'now'), and areas of methodological interest (e.g., the code 'continuum' was used for commentary about whether terms were represented appropriate poles of a shared domain of experience).

To enhance consistency across interviews, one member of our research team who was not involved in interviewing participants or questionnaire development completed the coding and primary analysis (JJT). Early in the process, our research team had detailed discussions about coding rules and conventions, the meaning and use of codes, and what kinds of text segments should and should not be coded. We discussed coded transcripts and made changes as needed throughout the process. We negotiated the validity of these decisions to consensus based on Sandelowski and Barroso's persuasive argument, following Eisner [27] and Morse [28], that the explicit process of negotiating validity to consensus may ensure more validity than demonstrating inter-rater reliability since "such techniques simply show that raters can, or can be made to, agree" [[29], p. 807].

Transcripts of cognitive interviews were coded according to the codebook described above. The vast majority of text in each of the transcripts was coded. Irrelevant side conversations were excluded from coding and analysis.

When coding was complete, we analyzed the data by running queries (in Atlas.ti) to examine the co-occurrences of each combination of (A) the 16 word pairs on the final version of the questionnaire and (B) positive or negative valence. These 32 combinations of word pair and valence serve as proxies for each of the 32 terms on the questionnaire. For each code-combination (term), we examined all of the quotations from the cognitive interviews--reading through the text, highlighting the relevant phrases, and removing quotations that did not include relevant content. Next we re-examined the relevant quotations and established summary descriptions for each of the terms and word pairs on the final questionnaire, based directly on participants' explanations in the cognitive interviews. Based on these descriptions, we selected representative/illustrative quotations for each of these terms. We also examined participants' responses to cognitive interview questions for the terms and pairs that were eliminated from the questionnaire. These findings are presented below.

## Results

The process of determining word pairs for the initial draft of the questionnaire is described in detail in Ritenbaugh et al. [12]. In forthcoming papers we provide details regarding quantitative data collection and analyses conducted to assess clinical meaningfulness and the psychometric performance of the questionnaire as administered to more than 600 participants (manuscript in preparation). Here, we report on the evolution of word pairs included on the Self-Assessment of Change questionnaire and we examine the meaning of terms based on the explanations given by participants in the cognitive interviews.

Twenty-eight cognitive interviews were conducted using five versions (v1-v5) of the questionnaire (Table 1). The first two versions each included 18 word pairs, and were tested in cognitive interviews: v1 with 12 participants and v2 with six participants. With 15 word pairs each, v3 and v4 were tested in three and four cognitive interviews, respectively. The final (v5) version of the questionnaire included 16 word pairs and was tested in three cognitive interviews before finalization. In the following sections, we discuss the derivation of word pairs included in the final version questionnaire by examining four categories of word pairs: those that remained stable across versions, those that were added to fill conceptual gaps identified by participants, those that evolved in response to participant feedback, and those that were eliminated over the course of cognitive interviews.

**Table 1:** Versions of the Self-Assessment of Change questionnaire

	Negative Pole	Positive Pole
**Version 1 (v1)**	Not sleeping well	Sleeping well
**18 word pairs**	Dull Senses	Vibrant Senses
**12 cognitive interviews**	Depleted	Vitalized
**Dated 11.19.09**	Suffering	Joyful
	Exhausted	Energized
	Scattered	Focused
	Powerless	Empowered
	Hopeless	Hopeful
	Unforgiving	Forgiving
	Isolated	Connected
	Life has no meaning	Life has meaning
	Have no Faith	Have Faith
	Overwhelmed	Resilient
	Closed-hearted	Open-hearted
	Broken	Healed
	Defined by my illness or problems	Not defined by my illness or problems
	Not on a spiritual path	On a spiritual path
	My body does not recover quickly	My body recovers quickly

**Version 2 (v2)**	Not sleeping well	Sleeping well
**18 word pairs**	Dull Senses	Vibrant Senses
**6 cognitive interviews**	Depleted	Vitalized
**Dated 2.8.10**	Suffering	Joyful
	Exhausted	Energized
	Scattered	Focused
	Powerless	Empowered
	Hopeless	Hopeful
	Unforgiving	Forgiving
	Isolated	Connected
	Life has no meaning	Life has meaning
	Sad*	Happy*
	Overwhelmed	Resilient
	Closed-hearted	Open-hearted
	Broken	Healed
	Defined by my illness or problems	Not defined by my illness or problems
	Anxious*	Calm*
	My body does not recover quickly	My body recovers quickly

**Version 3 (v3)**	Not sleeping well	Sleeping well
**15 word pairs**	Dull Senses	Vibrant Senses
**3 cognitive interviews**	Exhausted	Energized
**Dated 3.8.10**	Scattered	Focused
	Helpless	Empowered
	Hopeless	Hopeful
	Blaming*	Letting go*
	Isolated	Connected
	Very Sad*	Joyful
	Overwhelmed	Coping Well
	Closed-hearted	Open-hearted
	Broken	Whole*
	Defined by my illness or problems	Not defined by my illness or problems
	Anxious	Calm
	My body does not recover quickly	My body recovers quickly

**Version 4 (v4)**	Not sleeping well	Sleeping well
**15 word pairs**	Exhausted	Energized
**4 cognitive interviews**	Dull Senses	Vibrant Senses
**Dated 4.13.10**	Scattered	Focused
	Helpless	Empowered
	Overwhelmed	Coping Well
	Hopeless	Hopeful
	Blaming	Letting Go
	Closed-hearted	Open-hearted
	Isolated	Connected
	Depressed*	Joyful
	Anxious	Calm
	My body does not recover quickly	My body recovers quickly
	Broken	Whole
	Defined by my illness or problems	Not defined by my illness or problems

**Version 5 (v5)**	Not sleeping well	Sleeping well
**16 word pairs**	Exhausted	Energized
**3 cognitive interviews**	Dull Senses	Vibrant Senses
**Dated 4.26.10**	Scattered	Focused
**Finalized 5.10.10**	Stuck*	Letting Go
	Overwhelmed	Empowered
	Hopeless	Hopeful
	Blaming	Forgiving
	Closed-hearted	Open-hearted
	Isolated	Connected
	Depressed	Joyful
	Anxious	Calm
	My body does not recover quickly	My body recovers quickly
	Broken	Whole
	Defined by my illness or problems	Not defined by my illness or problems
	Unbalanced*	Balanced*

* indicates items changed from the previous version of the questionnaire

### Stable Word Pairs

Out of the sixteen word pairs that appear in the final (v5) version of the Self-Assessment of Change questionnaire, nine were included in v1 of the questionnaire and remain unchanged throughout the cognitive interview process (Table 2). The word pairs that remained stable throughout the cognitive interview process were well-understood by participants. Participants consistently said that the terms represented opposite ends of the same domain of experience, and with few exceptions, the explanations participants gave for the meaning of these terms were stable and consistent even if the participant did not view the particular domain as relevant to his/her individual experience. We illustrate this category by providing an exemplar stable word pair *(hopeless/hopeful)*, with a summary description and quotations illustrating how cognitive interview participants used each of the terms. (See Additional File 1 for descriptions and illustrative quotations of other stable word pairs.)

**Table 2:** Stable Word Pairs.

Negative	Positive
Not sleeping well	Sleeping well
Exhausted	Energized
Dull Senses	Vibrant Senses
Scattered	Focused
Hopeless	Hopeful
Closed-hearted	Open-hearted
Isolated	Connected
My body does not recover quickly	My body recovers quickly
Defined by my illness/problems	Not defined by my illness/problems

Word pairs that remained unchanged from the initial (v1) to final (v5) versions of the Self-Assessment of Change Questionnaire.

#### Hopeless/Hopeful

When explaining how they understood the word pair *hopeless/hopeful*, participants consistently described a particular orientation with the future (Table 3). The key aspect of the concept of 'hope' was not whether participants felt a sense of control over their ability to change their immediate circumstances so much as the extension of this agency into the future. In other words, in explaining the term 'hopeless,' participants not only described a sense of powerlessness over one's ability to change his/her experience of pain or grief, for instance, but they also extended this pessimism into the future. Participants described feeling resigned to the idea that 'things will never change' and that they fundamentally lacked options, or had exhausted all resources, for enacting any change in their experience. Interestingly, as in the evocative interviews [12], some participants in this research phase resisted labeling themselves as utterly 'hopeless,' saying that this was too extreme a characterization of their experience; nevertheless many identified as struggling with a broader sense of hopelessness. On the other hand, when explaining the meaning of the term 'hopeful,' participants reported feeling optimistic about the future and possessing not only an immediate sense of control over one's experience, but a broader confidence in the *potential *for successful outcomes in the future. Interestingly, many participants cited their CAM use as a specific source of hope, because it provided a new set of resources or treatment options where conventional solutions had been exhausted, thus providing a new sense of possibility for a better future. As with all the stable word pairs, participants consistently agreed that the terms represented opposite ends of the same domain of experience.

**Table 3:** Exemplar quotations of the word pair '*hopeless/hopeful*.'

Hopeless	Cog07	Hopeless would have to mean that there was no future; nothing I would do would make a difference.
	Cog18	I had hoped that the doctor would be a good stop for fixing this; I was hoping the doctor would fix it, and he didn't offer a resolution. So ... I kind of lost hope at that point, because I didn't know what else I was gonna do.
	Cog28	You better get used to where you're at, because nothing will ever change; like nothing can get better.
**Hopeful**	Cog07	Hopeful means that there is another day tomorrow, and you can make your life content and it could even be better the next day. Hopeful is having hope for the future.
	Cog16	Knowing that I can do something about it, through acupuncture, makes me feel hopeful.
	Cog21	Once I started seeing small changes and pretty dramatic ones through massage and yoga, then I obviously got much more hopeful.
	Cog25	Having hope and being able to move was so joyful and being able to talk with someone [CAM provider] who understood me, it was beyond any treatment and to feel like you have hope was immensely wonderful. I was feeling like so many doors opened at once.

### Added Word Pairs

In response to conceptual gaps identified by participants in cognitive interviews, two additional word pairs (*anxious/calm; unbalanced/balanced*) were added to later versions of the questionnaire.

#### Anxious/Calm

Early into the cognitive interviews, respondents spontaneously noted there was no item that adequately captured their experiences with stress or anxiety. After several participants emphasized that these concepts had been a central part of their illness experience and a site of important shifts in well-being, we were persuaded that adding a word pair to capture this domain of experience would strengthen the questionnaire.

The phrase "I was stressed" was well-endorsed in evocative interviews (phase 1b), but it was not included in the first version of the questionnaire because other phrases were endorsed more highly and investigators chose to limit the number of word pairs to eighteen in order to minimize the burden on respondents [12]. When we asked cognitive interview participants if they would use the term 'stressed,' several suggested that 'anxious' was more clear and precise. When asked to suggest a good opposite to 'anxious,' several spontaneous offered 'calm.' This word pair *(anxious/calm) *was added to v2 of the questionnaire and remained unchanged in the subsequent iterations of the questionnaire.

When asked to explain the meaning of these terms, participants referred to both the emotional and physical qualities of their experience (Table 4). Interestingly, a few participants characterized 'anxious' as a durable character trait (e.g., "I tend to be an anxious person") that can 'flare' at times of stress or suffering. On an emotional level, participants described 'anxious' as feeling worried, nervous, or 'stressed;' while on a physical level, they described the experience of a racing heart and the inability to relax the body and mind. Conversely, participants characterized 'calm' as an inner sense of being 'at peace' or 'at ease.' They describe feeling able to focus, concentrate, and control their inner experience. On a physical level, participants noted feeling comfortable, relaxed, and 'steady.'

**Table 4:** Exemplar quotations of the word pair '*anxious/calm*.'

Anxious	Cog09	At the time, several years ago, it was hard to not be anxious; it was hard not to worry and not to fret. I definitely had good days, but more often than not, I would be anxious, worrying, "Oh my gosh, I need to take care of that, what about this? What about that?"
	Cog15	Anxious? Oh boy. Never relaxing, never enjoying calm or quiet, because there was always a pounding in my head. There was no such thing as inner peace, nothing.
**Calm**	Cog22	I found myself feeling a lot calmer afterwards, and just a lot less like possessive of the way I wanted things to turn out, and it's not turning out that way, and I could just deal with it as it was.
	Cog25	Peaceful and steady and it has to do with walking a lot, like a rhythm that's steady and centered.
	Cog28	A sense of inner peace and an absence of fear.

#### Unbalanced/Balanced

Because there were several other 'whole person' [9,30] word pairs included in the questionnaire, one specifically referring to 'balance' was not added until the last iteration (v5) of the questionnaire, after several participants spontaneously noted that the questionnaire lacked a word pair to capture the overall sense of well-being or 'balance' they associated with their CAM use. While this gap was particularly salient for participants who had used yoga, tai chi and acupuncture, it was also apparent to those who described other terms as "too negative" or "too positive" and were searching for a way to signify a general shift to a better, more stable and comfortable state. Based on this feedback, we re-examined prior data and found that the phrases "I felt unbalanced" and "My life is balanced" had been moderately-endorsed in evocative interviews [12]. Based on these phrases, the word pair *unbalanced/balanced *was added before the last set of cognitive interviews and found to work well to capture this domain of experience. When asked to explain the meaning of these terms, participants described 'unbalanced' as the experience of extreme fluctuation in emotions or dyssynchrony between mind, body, and emotion. They characterized 'balanced' as feeling steady or calm in mind, body, and emotion. (See Table 5.)

**Table 5:** Exemplar quotations of the word pair '*unbalanced/balanced*.'

Unbalanced	Cog01	I was so focused as a hyperactive adult, that I couldn't see the forest for each tree I was confronting.
	Cog28	Like the emotional and physical body aren't working well together.
	Cog29	When you're at the extremes of emotion or even in extreme physical action.
**Balanced**	Cog16	I'm a hopeful person when I feel balanced.
	Cog29	You're ... more centered, more like there's not one thing that's just throwing you off.

### Evolving Word Pairs

Five word pairs on the Self-Assessment of Change questionnaire underwent revision based on data obtained during the cognitive interview process. As we described above, for each word pair on the questionnaire we asked participants whether the terms represented opposite ends of the same domain of experience. If participants answered in the negative, we encouraged them to suggest changes to one or both of the terms. Because the primary goal of this research phase was to refine the questionnaire, we were highly sensitive to participants' responses, which, in no small part, drove changes to the questionnaire. In addition, we asked participants how they interpreted each of the terms individually. Here, we were concerned with issues of consistency and stability in the meanings participants ascribed to terms. When we saw disagreement in participants' interpretations of terms, we made changes to the questionnaire. The specifics of these revisions are described below.

#### Blaming/Forgiving

**Figure 1:**
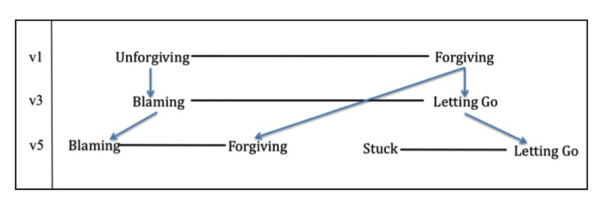
**Derivation of the word pairs 'blaming/forgiving' and 'stuck/letting go'**.

In the first round of cognitive interviews, participants were asked to evaluate a word pair consisting of the terms 'unforgiving' and 'forgiving.' In several instances, participants responded by questioning the referent of the item (e.g., "Unforgiving and forgiving of whom?"). Participants were unwilling to label themselves as 'unforgiving' unless it was qualified, such as 'unforgiving of myself.' Furthermore, in this first set of interviews several participants felt that 'forgiving' was 'too religious' and did not adequately characterize their range of experiences which, they noted, were more about not worrying, harboring resentments, grudges, or blaming others. When we asked these early participants to provide alternative terms, 'letting go' was suggested as a replacement for 'forgiving,' and 'blaming' for 'unforgiving' (Figure 1). When we tried these terms (*blaming/letting go*) as a word pair in a subsequent set of cognitive interviews, we found that, while these terms individually resonated with participants, they felt that the terms did not work well as opposite ends of the same domain of experience. In the course of additional interviews, 'forgiving' re-emerged as an appropriate opposite to 'blaming.' Interestingly, participants had no difficulty responding to the word pair *blaming/forgiving*, as they had to *unforgiving/forgiving*. The term 'blaming' captured participants' feelings of anger and frustration with oneself and with others in cases of illness and hardship. Furthermore, when combined with 'blaming,' the term 'forgiving' became easier for participants to interpret. Participants described 'forgiving' as a fundamental character trait, often challenged in the course of illness, characterized by compassion for the mistakes and faults of others and themselves (Table 6).

**Table 6:** Exemplar quotations of the word pair '*blaming/forgiving*.'

Blaming	Cog21	Blaming is ... when you hang on to an event and think, "if only I could have changed it" ...and then you explain all subsequent failure with that event.
	Cog27	That you want to blame people for everything that happens. ... you want to find a scapegoat; you want to find a reason that that happened.
**Forgiving**	Cog09	I allow myself to make mistakes, and when I do, I don't grind myself into the floor for it, and the same with other people.
	Cog13	Just being more merciful with myself and other people. Just letting things go, and not holding on to them. I feel like I am much more forgiving, actually.

#### Stuck/Letting Go

As noted above, while the term 'letting go' resonated with participants' experiences, it did not work well as an opposite for 'blaming.' In the course of additional cognitive interviews, participants suggested terms to capture the notion of 'hanging on' to something, not being able to let go or move on. The term 'stuck' emerged as an option and was paired with 'letting go' for the final set of cognitive interviews (Figure 1). Participants likened 'stuck' to 'being in a rut,' 'going around in circles,' or being unable to break out of a pattern that was not serving them well. In an earlier version of the questionnaire, the term 'stuck' emerged in relation to the terms 'powerless' and 'exhausted,' where participants spontaneously used the term to describe feeling unable or unmotivated to change a difficult situation.

Interestingly, participants in this final set of interviews suggested that we keep both word pairs (*blaming/forgiving *and *stuck/letting go*) as they captured different domains of their experience. Participants distinguished 'letting go' from 'forgiving' by explaining that the former emphasized a *process *of accepting one's situation, making peace with it, and moving on. It also implies *releasing *oneself and/or others from anger, blame, frustration and worry. In short, while *blaming/forgiving *involves one's relationship with others and with concepts of responsibility and fault (Table 6, above), the word pair *stuck/letting go *was more oriented toward one's inner struggle and acceptance (Table 7).

**Table 7:** Exemplar quotations of the word pair '*stuck/letting go*.'

Stuck	Cog29	Stuck means you can't break out of a certain pattern, or you just hold on to the same ideas, or you feel like you can't change your situation. Lack of power to do that.
**Letting Go**	Cog21	It's like making peace and realizing there's nothing I can do about it or not letting it rule my life.
	Cog23	Letting go is just almost like saying to yourself, "Hey, it's not your fault." You've been blaming yourself for something that was never your fault to begin with.
	Cog29	I see it as when you realize that you're holding on to something, or that you're stuck in a pattern, realizing that and just moving past that. Pretty much, by not necessarily reaching a resolution, but having your resolution be, "You know what, I need to let go."

#### Overwhelmed/Empowered

**Figure 2:**
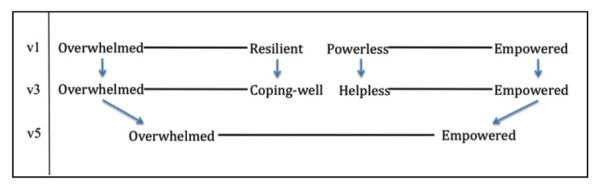
**Derivation of the word pair 'overwhelmed/empowered'**.

The initial version of the questionnaire included two word pairs (*overwhelmed/resilient *and *powerless/empowered*) that went through revisions and were combined into a single word pair (*overwhelmed/empowered*) in the final (v5) version of the questionnaire (Figure 2). Early in the cognitive interviews, we found that the term 'overwhelmed' strongly resonated with participants' experiences of feeling unable to manage or escape from intense emotional or physical demands. However, while 'resilient' fit the other end of the continuum for some participants, others resisted describing themselves this way and suggested that a broader population might not understand the term. 'Coping-well' was suggested as a more accurate and accessible opposite for 'overwhelmed' and was tested in the third round of cognitive interviews (v3). Here, some participants argued that 'coping-well' did not fully capture the essence of what it means to not be overwhelmed, which involves having the resources, ability, and/or willingness to confront an untenable situation. Despite its use in another word pair, several participants suggested that the term 'empowered'--implying that one possesses the confidence, knowledge, motivation to confront life's challenges--was a better opposite for 'overwhelmed' than either 'resilient' or 'coping-well' (Table 8). With regard to the term 'powerless' (originally paired with 'empowered'), participants in early cognitive interviews found the term too extreme a characterization of their experience and rejected the notion that they had "no power." In the second round of interviews, the term 'helpless' was accepted as a suitable alternative to 'powerless.' Nevertheless, in order to eliminate conceptual overlap and to minimize the number of word pairs on the questionnaire, researchers suggested that *overwhelmed/coping well *and *helpless/empowered *were similar enough that only one pair should be selected for the final questionnaire. Thus, because the terms 'coping-well' and 'helpless' had received more lukewarm responses from participants, the terms 'overwhelmed' and 'empowered' were combined into a single word pair. To verify this change, participants in the final set of cognitive interviews were asked whether *overwhelmed/empowered *worked better than the other word pairs that had been tried. Participants were satisfied that this word pair captured this domain of experience better than either of the other two.

**Table 8:** Exemplar quotations of the word pair '*overwhelmed/empowered*.'

Overwhelmed	Cog03	I was overwhelmed, spiritually, emotionally, physically, every one of these. I was totally through. I just didn't even want to get out of bed in the morning, sometimes.
	Cog07	[I was] overwhelmed because all the sudden there were so many things to get done: having surgery, recovering from surgery, having chemo; before that, making a will, and making a power of attorney, and talking to my kids about what's happening; and moving, and getting a place to live. It was huge.
	Cog15	When I was overwhelmed, it was overwhelmed with the pain, the pain controlled everything and I don't think I was able to escape from it.
**Empowered**	Cog01	Empowered would mean that beside, beyond energy, that I had the intellectual capacity and the ability to focus as a combination that would allow me to deal with the issues in life effectively.
	Cog14	The whole treatment made me feel empowered when I found a choice of something that does work. Something can work.
	Cog25	The way [my acupuncturist] talked to me was very empowering; about how things could change and about how they could be different. He didn't see it and say, "I can't solve this" and it was about solving, it was about the process of moving forward. I thought that was really powerful.
	Cog27	I think this is one of the few long-term effects of meditation... just do something simple and get a handle on your life. That's pretty empowering, because you realize you can handle a lot of different things.

#### Depressed/Joyful

**Figure 3:**
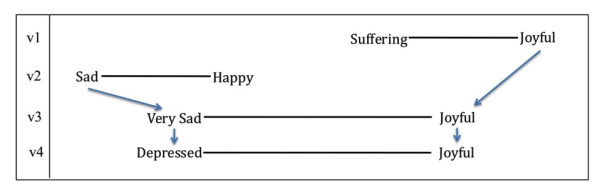
**Derivation of the word pair 'depressed/joyful'**.

The first version of the questionnaire included the word pair *suffering/joyful*. In cognitive interviews, participants expressed a strong distaste for the term 'suffering.' They resisted characterizing themselves this way and argued that 'suffering' was an overly excessive characterization of their experience with physical and/or emotional pain. The term 'joyful,' however, resonated with participants, although some described it as "too positive." Based on these reactions, we used the terms 'sad' and 'happy,' which had been suggested as more moderate replacements, in the next iteration (v2) of the questionnaire (Figure 3). Participants, however, were not content with these terms, describing them as 'too generic' and 'boring.' Several participants stressed that 'sad' was not far enough along the negative continuum and some even placed their marks off this side of the scale entirely. In response, 'joyful' was re-included due to the generally positive response it received in the first round of cognitive interviews and 'very sad' was substituted as the negative term in v3. Nevertheless, respondents suggested that 'very sad' still did not quite capture the essence of their feelings, which were deeper and more profound than 'sadness.' Looking back at earlier cognitive interviews, we found that participants had used the term 'depressed' to describe the emotional component of 'suffering.' In v4 of the questionnaire, we paired 'depressed' with 'joyful' and found that participants were satisfied with this word pair.

In their explanations of these terms, participants described a mood or emotional state that influenced many other areas of their lives (Table 9). Importantly, participants did not use the term 'depressed' as a clinical diagnosis; rather, they described an inability to enjoy life, friendships and the world around them, a dwelling in grief, sadness, and disappointment, and a lack of motivation to change the situation. In some cases, participants suggested that 'depressed' indexed a kind of emotional suffering or the emotional toll of physical suffering. On the other hand, participants suggested that the term 'joyful' broadly conveys a sense of happiness, hopefulness, and openness, and a kind of exuberant and creative energy. It implies an ability to enjoy relationships and life-experiences to their fullest, along with a sense of contentment, appreciation, and gratitude.

**Table 9:** Exemplar quotations of the word pair '*depressed/joyful*.'

Depressed	Cog23	Depressed is feeling unhappy, pretty much by yourself. ... Not being able to see the nice things that are around you. It can be a beautiful day, lots of beautiful flowers, cool breeze, anything. But ... you're still not going to see them feel them, acknowledge them.
	Cog27	When I feel a sense of depression, it relates to a lot of these questions on the negative side of things, hopelessness and overwhelmed, scattered, isolated, all those things lump together and make depression. It's physical as well. ... It's not as easy to pinpoint what it is, because it relates to so many other things.
**Joyful**	Cog17	I can see that as a sort of vibrancy, an ability to really experience the good times of life and enjoy the people you're with, and enjoy the things you do.
	Cog25	I felt so joyful that I felt inspired to do more, so it was kind of exponential. ... So good to start being able to live.

#### Broken/Whole

**Figure 4:**
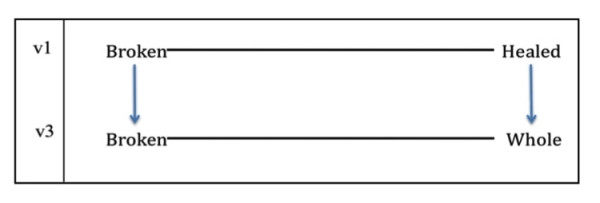
**Derivation of the word pair 'broken/whole'**.

In response to the word pair *broken/healed *in early versions of the questionnaire, several participants argued that the term 'healed' focused too narrowly on bodily wellness and did not adequately capture the 'whole person' quality of the term 'broken.' Participants described 'broken' as an emotional, physical, and spiritual concept that drew upon many of the other terms on the questionnaire, such as 'overwhelmed,' 'scattered,' and 'hopeless.' The term 'whole' replaced 'healed' in v3 of the questionnaire, and received a better response from participants (Figure 4). Whereas 'broken' suggested that the mind and/or body was not functioning properly, 'whole' carried a sense of overall wellness. Furthermore, the word pair *broken/whole *was better able to capture participants' emotional and spiritual experiences, essential for participants who did not suffer from a physical condition (Table 10).

**Table 10:** Exemplar quotations of the word pair '*broken/whole*.'

Broken	Cog07	My body was broken... A lot of things with [cancer] surgery make your body broken. It's cracked; it needs to be healed--that's the body. Broken could be frightened, could be like a broken spirit, and I'm sure I had days I felt that way.
	Cog13	Broken, to me, means ... there's no possibility of ever getting fixed again
	Cog21	I think when something happens to you like chronic back pain, you do feel like your body's not working properly, like something's broken. Was I a broken individual? No, I wouldn't say that, but I did say that I felt like my body wasn't like, fully functioning.
**Whole**	Cog09	[Despite] the traumas I have been through, I feel whole. I don't feel like I'm limping along spiritually or physically.
	Cog23	You're all together, you feel better.

### Eliminated Word Pairs

Four word pairs included on v1 of the Self-Assessment of Change questionnaire were eliminated over the course of cognitive interviews based on participant feedback. These word pairs were eliminated either because participants viewed them as redundant with another, more precise, word pair or because the definitions participants gave for the terms were unstable and inconsistent.

#### Depleted/Vitalized

This pair was eliminated from v3 of the questionnaire because interviewers noticed that participants were placing their marks on the line in almost exactly the same place as the word pair *exhausted/energized*. When interviewers asked about this, participants said that they were unable to determine a clear difference between the two word pairs and felt that they essentially measured the same domain of experience. In addition, some participants spontaneously asked interviewers to clarify the difference between *depleted/vitalized *and *exhausted/energized*. When interviewers asked respondents which pair they preferred, they overwhelmingly chose *exhausted/energized*, noting in several cases that the words 'depleted' and 'vitalized' are less common in everyday parlance.

#### Life has no meaning/Life has meaning

This pair was also eliminated from v3 of the questionnaire. Participants overwhelmingly said that 'life has no meaning' suggested that life might not be worth living--a sentiment that was far too extreme, even for those who had endured severe suffering. Several participants suggested that the term 'hopeless' better captured this aspect of their experience. At the other end of the continuum, many participants said that their lives had *always *had meaning, even during the worst of times.

#### Not on a spiritual path/On a spiritual path and Have no faith/Have faith

These two pairs aimed to address spiritual and/or religious experience, themes that emerged particularly strong among racial and ethnic minorities in Phase 1a and 1b of this research project [12] and in other studies of CAM use [10,31]. Nevertheless, these items were eliminated from v2 of the questionnaire because multiple participants expressed confusion and/or discomfort with the terms. Some felt that they were explicitly religious terms from a faith to which they did not ascribe, while others felt they were too ambiguous and open to interpretation. Others were confused by the terms altogether, asking for clarification from the interviewer. Although participants struggled to understand these word pairs in particular, cognitive interviews revealed that the underlying concepts of faith and spirituality were indexed in other word pairs included in the questionnaire (e.g., *hopeless/hopeful*, *closed-/open-hearted, isolated/connected*), as illustrated by the quotations below (Table 11).

**Table 11:** Sample quotations related to '*spirituality*' as captured by other word pairs.

*Hopeless*	Cog06	No trust, no belief in a higher power, or even, not just in God, but in people; just kind of dwelling on all the negatives, all the bad things. Hopeless would be another word for it.
***Hopeful***	Cog01	Now I feel more hopeful because I've been able to add a deeper spiritual dimension.

***Closed-hearted***	Cog09	Somebody who doesn't allow themselves to experience goodness, happiness, peace, on a spiritual level; just very closed off to that kind of thing. ... I didn't allow myself to experience those things on a spiritual level.

***Open-hearted***	Cog01	Open-hearted. Again, it gets back up into having faith, or being connected, and there, I never lost my open-heartedness, even in the worst time.

***Isolated/Connected***	Cog05	The more that I am fully present in each moment, I am more connected with Spirit. The more that you are present in each moment, you find everything you need to find, the joy, and everything that you need and you're connected with Spirit. The more you are connected with Spirit, there is no isolation.

### Usability Testing

To ensure usability [13], we investigated whether respondents understood the questionnaire's format and directions, and whether they were able to complete the questionnaire without difficulty. As described above, cognitive interview participants were asked a series of questions that yielded rich data on these issues.

#### Shared Domains of Experience

Cognitive interviews demonstrated that participants had little difficulty understanding that each word pair was intended to represent positive and negative poles of a shared domain of experience. We saw a willingness on the part of participants to consider each domain of experience independently, and despite our purposive recruitment of participants who had experienced shifts in well-being following CAM therapies, participants also showed a willingness to indicate no change (81 out of 441 total items on completed questionnaires; 18.4%) or even negative movement (24 items; 5.4%) on their questionnaires. As expected based on our sample, most items (328 items; 74.4%) showed positive movement on the scale.^i^

The instances in which participants struggled to complete individual word pairs were particularly meaningful to our research team. In cognitive interviews, participants generally indicated these word pairs did not well-represent endpoints of a shared domain of experience. In these instances, participants were asked to recommended alternative terms that would better represent the domain. These suggestions provided the foundation for the modifications to the questionnaire discussed in detail above.

#### Retrospective Pre-test Format

Several studies have shown that patients' may change their treatment goals, or primary reason for using CAM, over time [3,4]. Early in Phase 1 of this research project we also noticed participants reporting 'surprise' at unexpected changes in their lived-experience and shifts in their 'internal frame of reference' (e.g., "I never knew that I could feel like this" or "I never imagined that I could feel so much joy") [12]. This observation motivated our research team to develop the questionnaire using a retrospective pre-test design described above. According to Drennan and Hyde [[15], p. 700],

One major problem with self-report pre-test-post-test measures is that the [respondent] may reconceptualise the construct under investigation between the pre-test (time one) and the post-test (time two) [16]. This reconceptualisation may lead the [respondent] to evaluate the outcome under investigation from a different perspective at the post-test stage from the one he/she held at the pre-test stage. This change in perspective or 'internal frame of reference' is as a result of the student being exposed to the intervention between the pre-test and the post-test leading to a shift in his/her response [[32], p. 699].

The retrospective pre-test design, which was developed for the evaluation of learning outcomes in educational and training settings, has been shown to control for this 'response shift bias' and minimize both overestimation and underestimation of change [15,16,18,33,34].

To assess the effectiveness of this format, cognitive interview participants were asked to re-articulate the instructions aloud to the interviewer prior to completing the questionnaire. This data provides preliminary insight into whether respondents understood the retrospective pre-test format of the questionnaire and could locate their experience on the 100 mm visual analog scale for two points in time: 'now' and 'before' they began CAM treatment. Unlike the research and clinical contexts for which this instrument was designed (where both the 'before' and 'now' points will be unambiguous), our purposive sample asked individuals to identify a relevant time frame over which they would be doing the recall, and then to maintain that time frame while completing the questionnaire. We included individuals drawing on a range of recall periods, from several months to several years. Although a few participants needed verbal clarification, most participants indicated little difficulty recalling and indicating their experiences 'before' and 'now.'

Overall, cognitive interviews suggest that the retrospective pre-test format helped minimize response shift bias. Take the example of the word pair *closed-hearted/open-hearted*. In describing that they had 'always' considered themselves open-hearted but 'now' found themselves even more so, participants suggest that they would have located themselves at the far right (positive) end of the scale if they had completed a standard pre-test version of this questionnaire. Thus, a mark on the far right (positive) end of the scale in a post-test would suggest that participants had experienced *no change *in this domain of experience. Using the retrospective pre-test format, participants are able to integrate their *expanded sense of possibility *by placing their 'before' mark in positive territory while also allowing room to place their 'now' mark even further along the scale to indicate positive change.

#### Relevance of items to individual experience

As we describe elsewhere, the evocative interviews, in which participants ranked phrases based on relevance, allowed us to identify items for inclusion on the questionnaire that were *most relevant *to participants' individual experiences with CAM [12]. In revising the questionnaire, we focused on developing a comprehensive, non-redundant set of items that would represent the *full range *of patients' individual experiences. Because the questionnaire is designed for clinical and research contexts, in our instructions for completion we deliberately refrained from asking respondents to speculate about the linkages between interventions and outcomes, or tasking them with making causal attributions for the changes in their experience.

From a methodological perspective, however, we were interested in understanding whether participants had difficulty responding to items on the questionnaire if they did not perceive them as relevant to their individual experience. To examine this issue, participants were asked during the cognitive interview to articulate whether "the two endpoints on the scale somehow relate to your experience as you went through this change?" Participants were also asked to explain how they determined where to place their 'before' and 'now' marks on the scale. In cognitive interviews, participants said that 98 items (22% of the 441 total items on completed questionnaires) were unrelated to their individual experience. Nevertheless, participants were readily able to respond to these word pairs on the questionnaire itself. On the vast majority of these 'unrelated' items, participants indicated no change (both marks in the same place: 59 items, 60% of unrelated items) or very little change (both marks within 10 mm: 11 items, 12% of unrelated items). Participants skipped three items that they did not feel were relevant. Thus, out of 441 total item responses, participants only indicated change on 25 items (5.6% of total) they reported to be unrelated to the intervention they were referencing. From an overall trial perspective, this would present an acceptably small error in data.

#### Respondent Burden

Finally, although we recognize that many of the items on the final questionnaire reflect domains for which multi-item scales are available (e.g., hope, sleep, depression, control), we were sensitive to respondent burden associated with the administration of a battery of instruments. Our goal was to design a *comprehensive instrument *for measuring change over a broad range of domains of experience. The final version (v5) of the Self-Assessment of Change questionnaire includes 16 items, and preliminary data from cognitive interviews indicates that the respondent burden for this instrument was low. Respondents required only 10-15 minutes to complete the questionnaire and, out of 441 total items on completed questionnaires, only eight items (1.8%) were skipped. Subsequent quantitative data collection and analyses will address the psychometric properties of the questionnaire, including the construct validity of some of the single items when compared with existing multi-item scales, item responsiveness, between-item correlations, and other relevant metrics (manuscript in preparation).

## Discussion

In this paper, we describe the evolution of the Self-Assessment of Change questionnaire, which was designed to measure multi-dimensional shifts in well-being following CAM and other mind-body therapies. Verhoef et al. have observed the "growing recognition by CAM practitioners and researchers that the current array of outcome measures is not sufficient for use in CAM research and practice, as they do not cover the full spectrum of observed treatment effects" [[35], p. 2]. In fact, very few measures of patient-reported outcomes have been developed and evaluated in a CAM-specific patient population [36-38]. To fill this gap, databases and measures have been developed using a top-down approach that involves combining, selecting and altering items from extant questionnaires [e.g., [36,39]]. While this approach has the benefit of using items that have already been tested and validated in large populations, it primarily captures those outcomes that patients and providers seek and/or expect from CAM therapies.

In contrast, we have taken a decidedly different approach, focusing on the language patients use when they describe their first-hand experience with broad shifts in well-being following CAM therapies to develop a questionnaire that comprehensively measures patient-reported outcomes with CAM therapies from the ground-up. Our intent was to develop a patient-centered instrument to complement more specific measures of clinical outcomes (such as pain, disability, and function). While our questionnaire includes some outcomes that patients and CAM providers may seek and/or expect as a part of CAM therapy, this approach has also allowed us to identify and include changes that were unexpected and/or surprising to patients, and sometimes to practitioners, those we have termed *emergent outcomes*.

Several recent studies examining qualitative data on patient's experiences with CAM have identified categories comparable to those on our instrument. For example, in a qualitative analysis of responses to open-ended questions by 327 participants in five CAM clinical trials for back pain, Hsu et al. [11] identify a number of "unanticipated benefits" of CAM, including changes in hope, emotional states, body awareness, patterns of thinking that increase coping, overall health and well-being, and energy. Rugg et al. [40], examining the changes experienced by patients using acupuncture for medically unexplained physical symptoms, identify changes in physical, psychological and social dimensions of health, most notably increased physical/mental energy, sense of personal control, calm, and relaxation. In a qualitative synthesis of 26 studies of cancer patients' experiences with CAM, Smithson et al. [31] identify control (both empowerment and surrender), connection (with providers, of mind/body/spirit, and with a social group), pragmatic changes in well-being or quality of life, and (spiritual) transformation as key concepts emerging from this literature. Together, these findings enhance the face validity of our instrument.

This instrument is grounded in the lay language and first-hand descriptions of change that individuals experienced following the use of CAM therapies. At every stage of this research, we made deliberate efforts to remain true to the voices and experiences of our participants. Instrument development began with secondary analysis of first-hand descriptions of these changes (phase 1a), descriptions not bound by clinical terminology, but patients' own words. In the second step (phase 1b, evocative interviews), we asked a new sample to describe their experiences in their own words, but also to reflect and improve upon phrasing that emerged from phase 1a. Out of 38 interviews and 107 phrases, 18 word pairs were selected for the initial version of the questionnaire based on their relevance to patient experience, and a retrospective pre-test response format was designed in an effort to best represent the lay language and first-hand experience of participants' emergent and multi-dimensional shifts in well-being [12]. In phase 2 of this research, reported here, we 'closed the loop' by placing lay language and direct experience at the center of questionnaire revision and refinement. In a series of cognitive interviews, we systematically asked participants to evaluate the terminology on the questionnaire, to suggest alternatives and improvements, and to assess whether the word pairs were good representations of positive/negative endpoints of a single domain of experience. We used these data to revise and refine the questionnaire.

Several studies, especially those focused on practitioner-based CAM, have emphasized the centrality of the patient-provider relationship and experiences with health care delivery in patient-reported experiences with CAM [5,6,10,31,36,40]. For example, Smithson et al [31] identify *integration *and *polarization *(of CAM and biomedicine) as opposing concepts that affect patients' experience using complementary therapies in support of cancer care. While patients' individual experiences with CAM are likely affected by their positive and/or negative experiences of both CAM and biomedicine at the practitioner and organizational levels, our instrument focuses on the individual level of experience. While we do not include items expressly measuring changes in the patient-provider relationship or changes in health services delivery over time, participants indexed these underlying concepts in their descriptions of change to several domains included on the questionnaire (such as *hopeless/hopeful, my body recovers quickly/does not recover quickly; defined/not defined by my illness or problem*). We hope that our instrument can be used in research settings to inform issues related to the patient-provider relationship and health care delivery.

In this paper, we detail how this questionnaire was refined in an iterative process based on data from 28 cognitive interviews with individuals who experienced shifts in well-being following CAM and other mind-body therapies. We describe the derivation of the items on the final (v5) version of the questionnaire and provide explanations of the concepts being measured along with exemplar quotations from the cognitive interviews. Finally, we demonstrate that respondents understand the format and instructions for the questionnaire, and are able to complete it without difficulty.

The objective of this study was to design a questionnaire for use in clinical and research contexts with a broad population of patients using CAM and other mind-body therapies. CAM is a highly divergent collection of self-care and practitioner-based practices, however, and developing an instrument for broad use in a CAM setting requires prioritizing generalizability over specificity. Thus, we recognize that the terminology on the final questionnaire may not be the most evocative especially for particular subpopulations (e.g., racial/ethnic groups or patient populations using particular CAM modalities). To accommodate particular subpopulations, we invite clinicians and researchers to add or remove items from the Self-Assessment of Change questionnaire, but to refrain from changing any items they retain, as we are confident that they are representative of the experiences of CAM users, that respondents understand the terms in clear and consistent ways, and that the word pairs represent a clear and shared domain of patients' lived-experiences.

Although this instrument was designed and tested in a CAM-specific target population to fill a particular need in this community of practice, we believe that the instrument may also be useful in assessing multi-dimensional shifts in well-being beyond CAM, in a broader patient population. Further testing will be necessary to establish content validity in other populations.

Additional work with the instrument, including the instructions, will also be necessary before broadly applying it in epidemiologic settings where the event definition (i.e., 'before' and 'now' points in time) will be idiosyncratic to each participant, as was the case in the cognitive interviews. This presented minimal problems in the cognitive interviews described here due both to our recruitment processes and the ability of our interviewers to maintain consistency. In contrast, our data sets for psychometric evaluation are drawn from clinical trials and clinical settings where this is not a concern (manuscript in preparation).

## Conclusions

In this paper, we detailed a refinement of the Self-Assessment of Change questionnaire using an iterative process based on cognitive interviews with CAM users. We described the derivation of the 16 items on the final questionnaire and provided explanations and exemplar quotations of the measured concepts. Furthermore, we demonstrated that respondents could complete the questionnaire without difficulty.

This process of questionnaire development provides a model for the development of PRO instruments beginning with direct patient experiences. Although this instrument was designed and tested in a population that had used CAM therapies, it may be useful in assessing multi-dimensional shifts in well-being across a broader range of therapies. A network of researchers has formed to work with this instrument, sharing experiences and information. The questionnaire and access to the collaborative network is available through http://www.selfassessmentofchange.org.

## Competing interests

The authors declare that they have no competing interests.

## Authors' contributions

JJT carried out the qualitative data analysis and drafted the manuscript. KK coordinated the study, participated in data collection, and contributed to the interpretation of data and content of this manuscript. CR conceived of this study and was Principal Investigator, participated in its design and coordination, and contributed to the interpretation of data and content of this manuscript. AH contributed to the interpretation of data and to the content of this manuscript. CS participated in the design and implementation of the cognitive interviews, and interpretation of those interviews the questionnaire refinement. SJC led the design and implementation of the cognitive interview process, the development of the instrument format, and the final item decision-making process. All authors contributed critically to the final manuscript and approved the final version.

## Pre-publication history

The pre-publication history for this paper can be accessed here:

http://www.biomedcentral.com/1472-6882/11/136/prepub

## Supplementary Material

Additional file 1 **Stable word pairs**. This file provides summary descriptions for each of the nine 'stable' word pairs. This material is provided for readers interested in the specific meanings ascribed to each term by participants. We also provide quotations illustrating how cognitive interview participants used them in context. Click here for file

## Endnotes

^i ^A key issue with regard to applying patient-reported outcomes is determining what amount of change on an instrument indicates a clinically meaningful change to patients' experience or a change that affects clinical management on the part of providers [14]. A quantitative assessment of this issue is taken up in a forthcoming paper; however, from a qualitative perspective we were able to inquire as to *why *participants placed the 'before' and 'now' marks at particular points on the line. With very few exceptions, participants placing their marks at different places on the line verbalized a change in their lived-experience (whether positive or negative). Furthermore, most participants described the items where both marks were placed in the same point as either 'unchanged' over time or 'unrelated' to the specific CAM intervention they were referencing.
