# Supplementary material for: Developing a patient-centered outcome measure for complementary and alternative medicine therapies II: Refining content validity through cognitive interviews
Source: BMC Complement Altern Med. 2011 Dec 29;11:136. doi: 10.1186/1472-6882-11-136 (PMC3439682; doi:10.1186/1472-6882-11-136)
Supplement: Additional file 1 — Stable word pairs. This file provides summary descriptions for each of the nine 'stable' word pairs. This material is provided for readers interested in the specific meanings ascribed to each term by participants. We also provide quotations illustrating how cognitive interview participants used them in context. [file 1472-6882-11-136-S1.PDF]

Stable word pairs

Below, we provide summary descriptions for each of the nine word pairs that remained unchanged across versions of the Self-Assessment of Change Questionnaire. This material is provided for readers interested in the specific meanings ascribed to each term by participants. We also provide quotations illustrating how cognitive interview participants used them in context.

Not sleeping well/Sleeping well

During cognitive interviews, participants were asked what the terms 'sleeping well' and 'not sleeping well' had meant to them while they were completing that item on the questionnaire. Their answers reflected concerns with both the quantity and quality of sleep (Table S1). Participants said that 'not sleeping well' meant that they were not able to get enough sleep, either because they had trouble falling asleep or falling back to sleep after waking, or because they suffered from frequent waking. They also said that the quality of their sleep suffered: their sleep was restless, they were 'tossing and turning' or not sleeping soundly, or they woke feeling tired and unrested. Furthermore, several participants—like Cog16, in Table S1—emphasized that 'not sleeping well' had a negative effect in other experiential domains, making them feel generally pessimistic, hopeless, anxious, fearful, and so on. Conversely, while some participants said that 'sleeping well' meant that they would log seven or eight hours of sleep or 'sleep through the night,' most participants emphasized the *quality* of good sleep. For these participants, 'sleeping well' meant falling asleep quickly and easily, sleeping deeply and peacefully, and waking feeling rested, refreshed, and energized for the day.

Table S1. Exemplar quotations for the word pair *'not sleeping well/sleeping well.'*

**Not sleeping well**	Cog16	When I'm not sleeping well, sometimes I get so sort of situationally depressed, and it is a very hopeless situation, "Will I ever get a good night's sleep again? … How am I gonna sleep for the rest of my life?" So, I felt pretty hopeless.
	Cog28	It means either sleeping too light, or waking up over and over, or waking up and not being able to go back to sleep.
**Sleeping well**	Cog03	Now I could say I sleep like a log, except when I get the urges to go to the bathroom. So consequently, I'm sleeping better. Mental images don't keep me awake, it's a small physical thing that keeps me awake.
	Cog28	I go to sleep easily, and I wake up in the morning, and I feel calmed when I wake up.

Exhausted/Energized

When asked to explain what the word pair *exhausted/energized* meant to them, participants emphasized that both included physical and psychosocial components (Table S2). Participants described 'exhausted' as an all-encompassing, and in some cases disabling, physical and mental fatigue. In earlier interviews, they drew connections between the term 'exhausted' and the terms 'depleted' and 'suffering,' both of which were eliminated over the course of questionnaire revision because they overlapped excessively with 'exhausted.' The term 'energized,' on the other hand, connoted physical and emotional strength, resilience, and a certain motivation or eagerness to engage with the world.

Table S2. Exemplar quotations for the word pair *'exhausted/energized.'*

**Exhausted**	Cog01	Exhausted was hardly being able to get up out of bed, hardly being able to lift any weight, hardly being able to carry out the functions and activities of daily living, I just couldn't do anything.
	Cog07	I was exhausted during my [cancer treatment], because of what you go through, you're ill, you have surgery, you have chemotherapy. It's a physical issue and you're exhausted, physically. You're exhausted mentally, and you're exhausted emotionally.
**Energized**	Cog13	Energized just meant, inspired to get things done, and having the physical energy to go to work, and to have a social life and all those things.
	Cog25	You feel like you have the capacity to take on lots of tasks or to talk about things, to be there, and be involved, be active.

Dull Senses/Vibrant Senses

While participants certainly referred to physical acuity when defining the word pair *dull senses/vibrant senses,* many also highlighted the importance of one's awareness and appreciation

of sensory stimuli (Table S3). In explaining 'dull senses,' participants described 'feeling in a fog' or, like Cog03 in Table S3, "walking under water" and like Cog09, many described 'dull senses' as a lack of awareness and disengagement from the beauties and pleasures around them. Conversely, beyond sharpness in perception, participants described 'vibrant senses' as a broader experience of engagement, attentiveness, and enjoyment of their natural and social worlds.

Table S3. Exemplar quotations for the word pair *'dull senses/vibrant senses.'*

**Dull Senses**	Cog01	I couldn't count on my sense of … hearing, smelling, touching. I was just so seriously ill, that I was hardly functioning. I didn't have access to the input from my senses, at least at an effective level.
	Cog03	I felt like I was walking under water. … I really wasn't cognizant of what was going on. … It's sort of like walking in a nether-world. … Now I look back on it, I was lost in a nether-world. I just didn't see things clearly.
	Cog09	You just kind of plod through your day, you don't really notice that the sun feels warm or that food tastes good, that music is enjoyable, you're just kind of getting through everything.
**Vibrant Senses**	Cog07	[My] senses now … are amazingly vibrant. Having gone through a life-changing experience and looking at death, everything seems to be so new, and every little thing is exciting and I just enjoy it all, the sun, the moon, the stars, the clouds, the rain, everything.
	Cog18	There was just such a dramatic shift from that underwater, foggy feeling, and back to my sense of self and mental agility and clarity of thought. And that's what 'vibrant senses' refers to for me: clarity of thought, sense of self, awareness.

Scattered/Focused

Participants' descriptions of the meaning of the word pair *scattered/focused* emphasized the ability to concentrate and complete tasks (Table S4). In particular, participants stressed that 'scattered' implied a feeling of distraction or the disorganization of thoughts and actions. They drew connections between this term and 'overwhelmed.' Several participants described feeling like their thoughts or actions were 'all over the place.' On the other hand, 'focused' implied not only an ability to concentrate, but a broader sense of control over attention and action, a sense of being 'present' in the moment, and a feeling that one's thoughts and actions are organized, deliberate, or 'gathered.'

Table S4. Exemplar quotations from cognitive interviews for the word pair *'scattered/focused.'*

**Scattered**	Cog09	Before I started yoga classes, it was very difficult for me to follow through with a task, I'd start things and then I would either get easily overwhelmed or bored.
	Cog15	I was scattered because I couldn't focus on important things, I was focusing on the pain and the sorrow, what was happening inside of me, that I was not enjoying life and not focused on the importance of what was going on at the time.
	Cog18	I felt scattered because I just had no awareness, there was no mindfulness, and so I would just be going through my day … just flailing my hands wildly and occasionally I would hit something I was supposed to do. And I just didn't have any sense of purpose.
**Focused**	Cog07	I was really focused at the time of my experience, because I had to focus and everything else went away, so that's what I really did.
	Cog18	When I felt like I had [my problem] under control, I was able to get everything done that I wanted to get done, and I had a kind of presence and awareness of my work and I was more mindful.

Hopeless/Hopeful

When explaining how they understood the word pair *hopeless/hopeful,* participants consistently described a particular orientation with the future (Table S5). The key aspect of the concept of 'hope' was not whether participants felt a sense of control over their ability to change their immediate circumstances so much as the extension of this agency into the future. In other words, in explaining the term 'hopeless,' participants not only described a sense of powerlessness over one's ability to change his/her experience of pain or grief, for instance, but they also extended this pessimism into the future. Participants described feeling resigned to the idea that 'things will never change' and that they fundamentally lacked options, or had exhausted all resources, for enacting any change in their experience. Interestingly, as in the evocative interviews [1], some participants in this research phase resisted labeling themselves as utterly 'hopeless,' saying that this was too extreme a characterization of their experience; nevertheless many identified as struggling with a broader sense of hopelessness. On the other hand, when explaining the meaning of the term 'hopeful,' participants reported feeling optimistic about the future and possessing not only an immediate sense of control over one's experience, but a broader confidence in the *potential* for successful outcomes in the future. Interestingly, many participants cited their CAM use as a specific source of hope, because it provided a new set of

resources or treatment options where conventional solutions had been exhausted, thus providing a new sense of possibility for a better future. As will all the stable word pairs, participants consistently agreed that the terms represented opposite ends of the same domain of experience.

Table S5. Exemplar quotations of the word pair '*hopeless/hopeful.*'

**Hopeless**	Cog07	Hopeless would have to mean that there was no future; nothing I would do would make a difference.
	Cog18	I had hoped that the doctor would be a good stop for fixing this; I was hoping the doctor would fix it, and he didn't offer a resolution. So … I kind of lost hope at that point, because I didn't know what else I was gonna do.
	Cog28	You better get used to where you're at, because nothing will ever change; like nothing can get better.
**Hopeful**	Cog07	Hopeful means that there is another day tomorrow, and you can make your life content and it could even be better the next day. Hopeful is having hope for the future.
	Cog16	Knowing that I can do something about it, through acupuncture, makes me feel hopeful.
	Cog21	Once I started seeing small changes and pretty dramatic ones through massage and yoga, then I obviously got much more hopeful.
	Cog25	Having hope and being able to move was so joyful and being able to talk with someone [CAM provider] who understood me, it was beyond any treatment and to feel like you have hope was immensely wonderful. I was feeling like so many doors opened at once.

Closed-hearted/Open-hearted

In evocative interviews, the phrase 'I am able to love' received a very high number endorsements by participants [1]; however, in preliminary cognitive interviews, participants rejected the terms 'I am unable to love' and 'I am able to love.' Thus, the word pair *closed-hearted/open-hearted,* suggested by a study participant, was developed to represent this important concept that emerged from an earlier stage of research (Table S6). During cognitive interviews, participants described the term 'closed-hearted' as a self-imposed emotional, social, or spiritual isolation, with some calling it 'cold' and emphasizing a lack of compassion and empathy for others. On the other hand, participants characterized 'open-hearted' as an emotional connection and a feeling of joy, warmth, compassion, and love for others. Interestingly, many participants—including Cog01 in Table S6—described 'open-hearted' as a broader character trait that they continued to possess even during the worst part of their experience. Nevertheless, even among

those who said they had "always" been open-hearted, some said that this quality had been further enhanced as part of their broader shift in well-being.

Table S6. Exemplar quotations for the word pair *'closed-hearted/open-hearted.'*

**Closed-hearted**	Cog11	Closed-hearted for me just meant kind of shutting myself down emotionally and … not being very sympathetic or compassionate to situations.
	Cog15	And when you're in physical pain, you don't really have empathy for anything else or anybody else.
	Cog21	It means closing yourself to anything that might hurt you, being impermeable, not letting yourself feel things, good or bad, to protect yourself.
**Open-hearted**	Cog09	Sometimes it can hurt to be open-hearted, it can be a little painful, but … you can even learn to enjoy and accept that.
	Cog13	I was thinking how much more open-hearted I am now; especially with other people, I'm very compassionate with people, I listen to them, and care about them.
	Cog18	Interestingly, open-heartedness was something that came out of the Reiki that I wasn't looking for, because one of the things that I felt during that initial transformation was this open-hearted joy, even though I said I never use that word.

Isolated/Connected

When asked to explain what the terms 'isolated' and 'connected' meant to them, participants emphasized both the degree to which they were open and accepting of offers of social support from others, and the degree to which they received that support (Table S7). They described 'isolated' as the experience of lacking social support, feeling alone, rejected and even unwelcome. At the same time, participants—like Cog05 in Table S7—also described deliberately isolating themselves by rejecting the help and company of others and pushing others away, often as a result of physical and/or emotional suffering. Conversely, participants characterized 'connected' as feeling supported and accepted, and being open to relationships with friends, family, community, support groups, and the spiritual world. Like 'open-hearted,' several participants characterized 'connectedness' as a stable character trait that was, if anything, enhanced as part of their shifts in well-being.

Table S7. Exemplar quotations for the word pair *'isolated/connected.'*

**Isolated**	Cog05	I did feel more isolated then and I was more isolated then, because there were just certain things I just didn't want people to know what was going on in my life, so I isolated myself for that reason.
	Cog14	You feel like nobody else has this [problem] …So you feel a little isolated when you don't realize it. You go through that, "Why me?"
	Cog23	Isolated means being pretty much by yourself. Being around other people, but those other people not really accepting you, understanding you, maybe welcoming you. It's very difficult, because there can be people that are nice to you, but then you feel like you're really not part of the group.
**Connected**	Cog15	Well, connected means I'm open to relationships, friendships, responsibilities, other people's problems, I can deal with.
	Cog23	Connected actually means being accepted by others, to the point where you actually feel comfortable and not feeling like you just have to be polite all the time to be able to get along with people.

My body does not recover quickly/My body recovers quickly

With regard to the pair *my body does not recover quickly/my body recovers quickly*, participants certainly emphasized their experiences with length of illness, pace of recovery, and their overall sense of ease in recovering from illness or injury. Importantly, however, participants also indexed a broader sense of the body as *ill versus healthy*, as well as a more general experience of *vulnerability versus resistance* to illness or injury, often vis-à-vis perceived immune function (Table S8). In defining the phrase 'my body does not recover quickly,' many participants described a generalized sense of susceptibility to illness or injury, an inability to prevent or recover from illness or injury, as well as the persistence of aches, pains, and other symptoms of illness—often as the result of cumulative insults such as pain, stress, grief, exposure to toxicity, and so on. On the other hand, in explaining what the phrase 'my body recovers quickly' meant to them, participants described a strong sense of confidence and trust in their body's ability to resist and recover from illness or injury.

Table S8. Exemplar quotations for the pair *'my body does not recover quickly/my body recovers quickly.'*

**My body does not recover quickly**	Cog03	My pain, my grief manifested itself into something physical in my body. So [my body] did not recover quickly, because [my daughter] died in 2000, and the kidney thing started in 2002. So, all of this illness and toxicity had built up in my body.
	Cog08	When I usually get a sore throat, I get a cold the next day, then the sore throat's gone in a couple. I had a sore throat for twelve days.
	Cog18	I had one year when I was just ill, repeatedly. I had colds, had flus, like three or four times during the year. And then, I also hurt my back, which was really strange for me. So I had muscle aches and it took so long to remedy that.
**My body recovers quickly**	Cog06	So I have an immune system now, I can be around people who are really sick and not get sick, you know, but I have to work at healing my body, because that's a lot of damage.
	Cog09	I'll have the occasional lower backache, but, you know, a few minutes under a warm shower and I'm good to go. Or I'll have a headache from my jaw, and I'll take some [medicine] or put a heat pack on, then I feel better. It's definitely not a chronic aches and pains kind of thing.
	Cog18	I didn't get sick as often, or I didn't feel like it. I didn't feel like I got injured as often. So it wasn't even a question of my body recovering quickly, it was … just to being healthy.

Defined by my illness or problems/Not defined by my illness or problems

When explaining what the terms 'defined my by illness or problems' and 'not defined by my illness or problems' meant to them, participants emphasized the degree to which their illness was central to their personal and social identity (Table S9). For many participants, being 'defined by my illness or problems' meant that one was focusing on one's illness or problems so much so that they had become a primary part of one's self-identity and began to dominate his or her thoughts, behaviors, and life. In some cases—like Cog07 in Table S9—participants emphasized that assuming an illness identity was a key aspect of enduring intensive treatment for serious illness. Conversely, being 'not defined by my illness or problems' meant that participants emphasized that distinction between an illness or problem that required management and their broader sense of self. In this case, participants stressed the importance of *not* allowing health issues to define or control one's life or identity.

Table S9. Exemplar quotations for the pair *'defined by my illness or problems/not defined by my illness or problems.'*

**Defined by my illness or problems**	Cog06	I'd been a binge drinker and in the end my binges just got closer and closer together, and so that was a large part. I lived to drink, that's who I was.
	Cog07	You are so focused... on the healing, or the experience, because you are at the doctors' and you're at the chemo room, and you're in the bathroom and … that's all you do is think of yourself, and so does everybody else think about you too.
	Cog11	Of course dealing with HIV and all of the stigmas … was consuming a large part of my life and thoughts and everything at the time.
**Not defined by my illness or problems**	Cog01	I have my physical limitations, but I also have my means of dealing with them, therefore, they don't define me.
	Cog06	I'm not totally defined by my illness as much as by my recovery, not defined by my problem, I do always keep in my mind that I'm an alcoholic, but I'm not defined by it. You can't get close to me or become my friend without me, at some point telling you that. It's important, but it's not all that I am anymore.
	Cog13	I just simply don't feel defined by illness problems anymore. I just don't, I don't feel like that is who I am… I know that we're much more than the body, and I don't want to limit myself in that way, define myself in that way. I just don't believe it anymore.

References (Additional File 1, only)

1. Ritenbaugh C, Nichter M, Nichter M, Kelly K, Sims C, Bell I, Casteneda H, Elder C, Koithan M, Sutherland E *et al*: **Developing a Patient-Centered Outcome Measure for Complementary and Alternative Medicine Therapies: I. Defining Content and Format**. *BMC Complementary and Alternative Medicine* 2011, **11**:135.
